# A Novel Regulatory Role for RPS4Y1 in Inflammatory and Fibrotic Processes

**DOI:** 10.3390/ijms26136213

**Published:** 2025-06-27

**Authors:** Karosham D. Reddy, Senani N. H. Rathnayake, Sobia Idrees, Fia Boedijono, Dikaia Xenaki, Matthew P. Padula, Maarten van den Berge, Alen Faiz, Brian G. G. Oliver

**Affiliations:** 1 School of Life Sciences, Faculty of Science, University of Technology Sydney, Ultimo, NSW 2007, Australia; senani.rathnayakemudiyanselage@uts.edu.au (S.N.H.R.); sobia.idrees@uts.edu.au (S.I.); fia.boedijono@uts.edu.au (F.B.); matthew.padula@uts.edu.au (M.P.P.); alen.faiz@uts.edu.au (A.F.); 2 Respiratory Cellular and Molecular Biology Group, Woolcock Institute of Medical Research, Macquarie University, Macquarie Park, Sydney, NSW 2113, Australia; dia.xenaki@woolcock.org.au; 3 Groningen Research Institute for Asthma and COPD, University Medical Centre Groningen, University of Groningen, 9712 CP Groningen, The Netherlands; m.van.den.berge@umcg.nl

**Keywords:** Y-chromosome, inflammation, fibrosis, asthma, CRISPR-Cas9, RNA-seq, sex differences

## Abstract

Asthma is a chronic inflammatory respiratory disease well-known to demonstrate sexual dimorphism in incidence and severity, although the mechanisms causing these differences remain incompletely understood. *RPS4X* and *RPS4Y1* are X and Y-chromosome-linked genes coding ribosomal subunits previously associated with inflammation, airway remodelling and asthma medication efficacy. Particularly, RPS4Y1 has been under-investigated within the context of disease, with little examination of molecular mechanisms and pathways regulated by this gene. The ribosome, a vital cellular machinery, facilitates the translation of mRNA into peptides and then proteins. Imbalance or dysfunction in ribosomal components may lead to malfunctioning proteins. Using CRISPR-Cas9 knockout cellular models for RPS4Y1 and RPS4X, we characterised the function of RPS4Y1 in the context of the asthma-relevant processes, inflammation and fibrosis. No viable RPS4X knockouts could be generated. We highlight novel molecular mechanisms such as specific translation of IL6 and tenascin-C mRNA by RPS4Y1 containing ribosomes. Furthermore, an RPS4Y1-centric gene signature correlates with clinical lung function measurements, specifically in adult male asthma patients. These findings inform the current understanding of sex differences in asthma, as females do not produce the RPS4Y1 protein. Therefore, the pathologically relevant functions of RPS4Y1 may contribute to the complex sexually dimorphic pattern of asthma susceptibility and progression.

## 1. Introduction

The translation of mRNA to a polypeptide chain is essential in protein synthesis, where mRNA transcribed from DNA is converted into functional proteins. Ribosomes conduct this process. Ribosomes comprise four ribosomal RNAs (rRNAs) and multiple ribosomal proteins (RPs) [1], and these ribosomal protein subunits are differentially expressed across various tissues [2]. Dysregulated ribosome formation can affect processes such as cell cycle progression, immune signalling and development via extra-translational functions of RPs [2,3]. Studies show that RPs, rather than rRNAs, are critical for efficient ribosome assembly and mRNA translation.

The RPS4 ribosomal protein is an evolutionarily conserved small subunit involved in mRNA binding [4]. This gene is commonly found on autosomes in vertebrates, except in mammals, where X-chromosome (*RPS4X*)- and Y-chromosome (*RPS4Y1*)-linked versions exist [4,5]. A duplicated version of *RPS4Y1* called *RPS4Y2* exists, but it demonstrates precise expression in the testes and germ cells [6,7], whereas *RPS4X* and *RPS4Y1* are constitutively expressed [7]. Importantly, all previously studied primate lineages express *RPS4Y1* and *RPS4X*, implying a vital contribution to development and regular cellular function [8].

RPS4X and RPS4Y1 are 263 amino acids in size but share 92.8% amino acid homology [5], with 19 amino acid substitutions [5,9,10]. Lopes et al. identified eleven non-conserved amino acid substitutions [7]. These substitutions alter the chemical properties of specific amino acid residues, leading to a variation in the chemical properties between RPS4X and RPS4Y1. The consequence of this difference remains poorly understood, although it is hypothesised to cause differential functions [11]. RPS4X demonstrates complete homology with the X-chromosome-linked mouse RPS4 gene [9], indicating that the RPS4X structure has been conserved whilst RPS4Y1 has undergone mutations causing a divergence from RPS4X. Therefore, as males carry both X and Y chromosomes, they express two distinct ribosomes, while females only produce RPS4X-containing ribosomes. Any functional differences between RPS4X and RPS4Y1 may result in sexual dimorphism at the translational level between males and females. Amino acids conserved between RPS4X and RPS4Y1 in humans cluster at the N-terminal domain, illustrating this region’s importance for ribosomal interactions [7]. Despite amino acid substitutions, initial studies concluded that these proteins carry essential functions in translationally active ribosomes [5,11]. These findings demonstrate ribosomes contain either RPS4X or RPS4Y1 but do not explore whether RPS4Y1-containing ribosomes carry a unique function.

Many human diseases demonstrate sexual dimorphism in development, progression and prognosis. Asthma is a chronic inflammatory respiratory disease that culminates in variable airflow in patients. It affects nearly 300 million people worldwide [12]; yet, the exact cause of asthma remains unknown. Importantly, asthma demonstrates a sexually dimorphic pattern of susceptibility and severity [13]. In childhood, males have both an increased incidence and worse asthma outcomes [14]. However, females show greater susceptibility to asthma in adulthood and present with a higher hospitalisation rate [15]. There are several factors proposed to contribute to the complex interaction between biological sex and asthma, such as sex hormones, physiological differences, and the sex chromosome complement [16].

Gene expression imbalances between RPS4X and RPS4Y1, due to the sex chromosomes, have garnered attention as a critical biological factor contributing to disease processes [17,18]. RPS4X has been implicated in a worse prognosis in various cancer types [19,20,21], which is likely linked to RPS4X regulation of cell proliferation [19]. RPS4Y1 has been less widely studied but has been identified to be dysregulated in asthma [22]. Further, RPS4Y1 modulates the response of patients to oral corticosteroids [23] (an essential treatment for asthma patients), modulation of the inflammatory response [24] and fibrotic processes [25]. Notably, these processes are hallmark features of asthma, highlighting a potential contribution of RPS4Y1 to asthma pathology.

No studies thus far have clearly examined the molecular mechanisms regulated by RPS4Y1. This study aims to explore and characterise the regulatory role of RPS4Y1 for inflammatory and fibrotic processes relevant to asthma. Through a multi-omics approach, we highlight that RPS4Y1 functions via distinct and variable mechanisms to regulate protein production. We demonstrate that RPS4Y1 modulates the production of inflammatory factors and critical extracellular matrix proteins, affecting cell adhesion and migration. We identify the potential for RSP4Y1 to preferentially increase the translation of IL6 and tenascin-C mRNA. These data generate an RPS4Y1-specific gene signature that correlates with asthma severity in male patients as measured by lung function. Importantly, our study sheds light on the novel regulatory function of RPS4Y1. As females do not express RPS4Y1, its pathologically relevant functions may contribute to sex differences in asthma susceptibility and progression.

## 2. Results

### 2.1. RPS4Y1 Expression Is Differentially Regulated in Asthma

**Figure 1:**
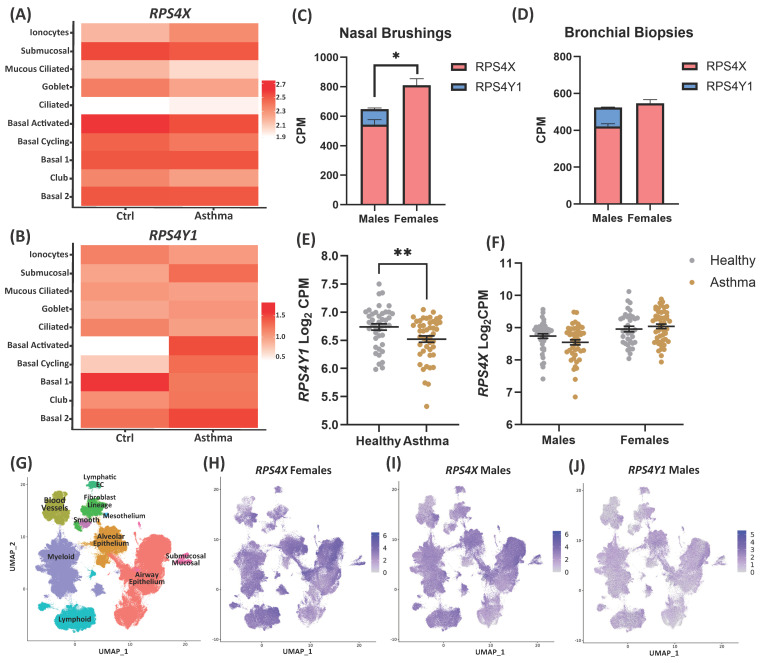
Gene expression of RPS4Y1 and RPS4X at a single cell level, bronchial biopsies and nasal brushings. Heatmaps of single-cell sequencing expression of RPS4X (**A**) and RPS4Y1 (**B**) across multiple cell types in healthy controls (ctrl); n = 4 female/7 males, and asthma; n = 2 female/7 male patients. Dark red represents higher expression, and white equals no expression. (**C**) nasal brushing; n = 101 females/89 males and (**D**) bronchial biopsy; n = 85 females/88 males of RPS4X (pink) and RPS4Y1 (blue) expression as counts per million (CPM). Data is analysed by fitting a mixed effects model. (**E**) RPS4Y1 expression as log_2_ CPM in healthy and asthmatic male bronchial biopsies. (**F**) RPS4X expression as log_2_ CPM in bronchial biopsies from healthy and asthmatic patients of both sexes. Grey represents healthy patients, and gold represents asthma patients. (**C**–**F**) data presented as the mean ± SEM. (**E**,**F**) is analysed by unpaired parametric *t*-test, statistical significance is indicated by * *p* < 0.05 and ** *p* < 0.01. UMAP of the identified cell types of single-cell RNA sequencing using data from the human lung cell atlas. (**G**) UMAP of defined cell locations, RPS4X expression in females (**H**) and males (**I**), (**J**) RPS4Y1 expression in males. Purple indicates higher expression, and white indicates lower expression.

To explore the expression profile of *RPS4Y1* and *RPS4X*, we investigated both at a single-cell level and in the lungs of healthy and asthmatic patients. The heatmaps presenting the expression of *RPS4X* (Figure 1A) and *RPS4Y1* (Figure 1B) indicate that their expression changes with asthma in a cell-specific pattern. Of note, *RPS4X* is most highly expressed in ‘basal-activated’ cells, with a similar expression level between asthma patients and healthy controls. *RPS4Y1* showed negligible expression in ‘basal-activated’ cells from healthy subjects but was significantly upregulated in these cells in asthmatic patients. As such, *RPS4Y1* expression demonstrates cell-specific responsiveness in asthma patients. UMAP analysis supports functions for these genes in basal cells. *RPS4X* is more widely expressed at higher levels than RPS4Y1, although both are expressed highly in alveolar and airway epithelium (Figure 1G–J).

Following the observations at a single-cell level, we expanded our analysis to the respiratory tract. We explored whether the combined expression of *RPS4X* and *RPS4Y1* in males equalled the expression of *RPS4X* from both X-chromosomes in females. In nasal brushings, combined *RPS4X* and *RPS4Y1* expression in males was lower than *RPS4X* expression in females (Figure 1C). However, in the bronchus, the expression levels of combined *RPS4X* and *RPS4Y1* in males were equal to *RPS4X* expression in females (Figure 1D). Importantly, in both nasal and bronchial samples, *RPS4X* expression in males was magnitudes greater than *RPS4Y1* expression. Finally, we confirmed in bronchial biopsies that *RPS4Y1* is downregulated in asthmatic males versus males without asthma (Figure 1E), but RPS4X demonstrated similar expression in males and females with asthma compared to healthy controls (Figure 1F). These results indicate a disbalance in the expression profiles of *RPS4Y1* and *RPS4X*.

### 2.2. RPS4Y1 Regulates Inflammation, Cell Attachment, and Migration

**Figure 2:**
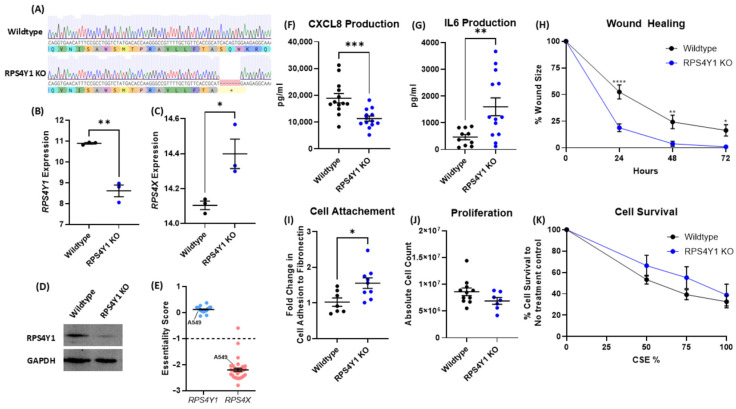
Characterisation of RPS4Y1 knockout cells (blue dots) compared to wildtype cells (black dots). (**A**) Representative chromatogram of wildtype and RPS4Y1 KO genome sequencing with codon translation to amino acid sequence underneath. Asterisk (*) = stop codon. Log_2_ counts per million (CPM) expression of *RPS4Y1* (**B**) and *RPS4X* (**C**) was measured by RNA-sequencing. (**D**) Representative western blot image confirming RPS4Y1 KO. (**E**) Essentiality score for *RPS4Y1* and *RPS4X* in multiple lung cell lines, with A549 cells identified; score < −1 indicates the gene is essential for survival. CXCL8 (**F**) and IL6 (**G**) production was measured in cell-free supernatant after 24h stimulation with TNFα (10 ng/mL) by ELISA. (**H**) The size of a scratch wound was measured every 24 h for three days with incubation in growth medium. Wound closure was measured as a percentage compared to the initial wound size at zero hours. (**I**) Cell attachment to fibronectin was measured after 1 h of incubation. (**J**) Proliferation was measured after 96 h of incubation in growth medium by manual cell counting. (**K**) Percentage cell survival compared to no treatment control (y-axis) was determined by an MTT assay after 24 h cigarette smoke extract (CSE) exposure. All data are presented as the mean ± SEM. (**H**,**K**) analysed by two-way ANOVA with Sidak correction for multiple comparisons testing. All other analyses were analysed by unpaired parametric t-tests. Statistical significance is represented by * *p* < 0.05, ** *p* < 0.01, *** *p* < 0.001 and **** *p* < 0.0001.

The UMAPs (Figure 1G–J) highlight *RPS4Y1* expression in alveolar and airway epithelial cells. Considering the alveolar-focussed expression of *RPS4Y1* and *RPS4X*, we used the alveolar basal epithelial cell line (A549) to create three unique RPS4Y1 knockout (KO) cell lines using CRISPR-Cas9. A549 cells are a male-derived cell line, providing an appropriate model to explore the expression of both X- and Y-chromosome-linked gene expression. These KO cell lines were characterised by changes in the inflammatory response, fibrotic processes, and the regulation of cell death. Successful RPS4Y1 KO via a frame-shift mutation was confirmed by genome sequencing (Figure 2A; Supplementary Figure S1), *RPS4Y1* gene expression (Figure 2B) and western blot (Figure 2D). A faint RP4SY1 protein band is observable in all knockout cell lines (Supplementary Figure S2), likely caused by cross-reactivity between the anti-RPS4Y1 antibody and RPS4X protein. Nonetheless, genome sequencing, RNA-sequencing, and protein immunoblotting demonstrate RPS4Y1 knockout. *RPS4X* gene expression significantly increases in RPS4Y1 KO cells (Figure 2C). This highlights a specific regulatory feedback mechanism between RPS4Y1 and RPS4X, where RPS4X production increases to compensate for the loss of RPS4Y1. Despite repeated attempts, it was not possible to generate RPS4X KO cell lines. An analysis of the online DepMap dataset revealed RPS4X is essential for cell survival, as indicated by an ‘essentiality’ score less than negative one (Figure 2E).

The immunoregulatory contribution of RPS4Y1 was assessed by stimulating cells with TNFα for 24 h. In RPS4Y1 KOs, CXCL8 production significantly decreased (Figure 2F), whilst IL6 production significantly increased (Figure 2G). The cells were also stimulated with IL-1β and TGF-β1 (two other pathologically relevant inflammatory cytokines), where CXCL8 was decreased and IL6 was increased in RPS4Y1 KOs versus wildtype (Supplementary Figure S3). This highlights an essential role for RPS4Y1 in regulating these cytokines.

RPS4Y1 KO cells demonstrated a significantly faster wound closure rate than wildtype cells (Figure 2H). We investigated cell attachment and cell proliferation to explore possible factors contributing to this observation. RPS4Y1 KOs reported increased cellular adhesion to fibronectin (Figure 2I), whilst no difference in proliferation by wildtype cells is noted (Figure 2J). Combined, these results indicate that increased adhesion promotes increased migration of RPS4Y1 KO cells, leading to a faster rate of wound closure. No change in cell survival to cigarette smoke extract (CSE) was observed (Figure 2K).

These data demonstrate that RPS4Y1 has a complex contribution to regulating asthma-relevant disease processes of inflammation, cell migration and adhesion, and changes to the extracellular matrix.

### 2.3. RPS4Y1 Differentially Regulates CXCL8 and IL6 Expression

**Figure 3:**
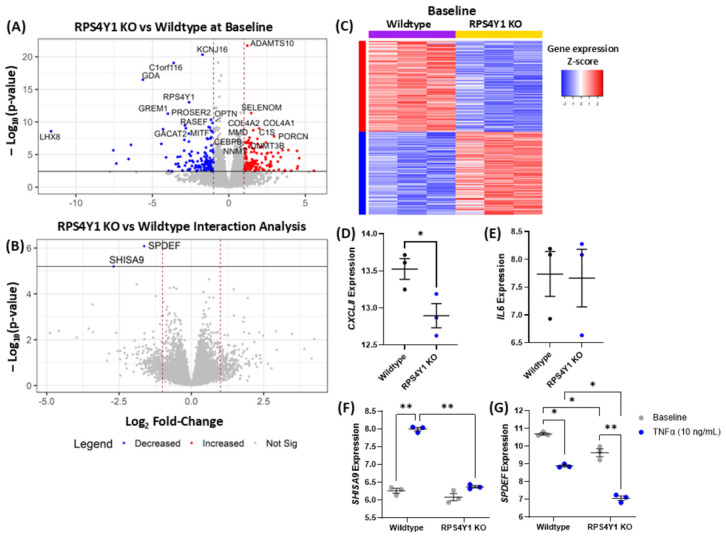
Differentially gene expression analysis of RPS4Y1 knockout vs. wildtype cell lines. Volcano plots of −log_10_(*p*-value) against log_2_fold-change in differentially expressed genes (DEGs) at baseline (**A**) and when KO and wildtype cell lines are stimulated with TNFα (10 ng/mL) for 6 h minus baseline DEGs (**B**). The horizontal black line represented a false discovery rate < 0.05; dotted red vertical lines indicate log_2_fold-change > |1.0|. Red dots indicate significantly upregulated genes and blue dots represent significantly downregulated genes in RPS4Y1 KOs. (**C**) Semi-supervised heatmap of DEGs at baseline between wildtype (purple) and RPS4Y1 KO (yellow) cell lines. Red indicates increased expression, and blue indicates reduced expression. Log_2_ counts per million (CPM) expression of *CXCL8* (**D**), *IL6* (**E**), *SHISA9* (**F**), and *SPDEF* (**G**) in wildtype and RPS4Y1 KO cells. Grey dots represent baseline levels; black and dark blue dots represent TNFα (10 ng/mL) stimulated samples. (**A**–**E**) Statistical significance was determined through differential gene expression analysis with multiple comparisons correction completed using the Benjamini–Hochberg method. (**F**,**G**) Two-way ANOVA completed statistical analysis with Tukey’s correction for multiple comparison testing. (**D**–**E**) Data are presented as the mean ± SEM. Statistical significance is represented by * *p* < 0.05, ** *p* < 0.01. n = 3 for all analyses.

Now that a functional effect of RPS4Y1 had been established, we conducted RNA-sequencing to determine whether knocking out RPS4Y1 significantly alters gene expression. Sequencing was conducted after 6 h of incubation with or without TNFα stimulation. At baseline (Figure 3A), RPS4Y1 KO cells demonstrated 125 significantly increased genes (FDR < 0.05 and log_2_FC > 1.0) and 139 significantly downregulated genes (FDR < 0.05 and log_2_FC < −1.0). An interaction analysis was completed, comparing genes differentially regulated between wildtype and KO cell lines after TNFα stimulation. Interaction analysis revealed no significantly upregulated genes, whilst *SHISA9* and *SPDEF* were the only genes downregulated (Figure 3B). This means they are relatively less expressed in RPS4Y1 KOs than wildtype cells after TNFα stimulation. *SHISA9* expression is significantly increased in wildtype cells upon TNFα stimulation, but this is lost in RPS4Y1 KO cells (Figure 3F). *SPEDEF* is an integral immunoregulatory and goblet cell differentiation factor in airway diseases [26]. This gene demonstrates reduced expression in RPS4Y1 KOs at baseline and a greater decrease in expression in knockout cells after TNFα stimulation (Figure 2G). The heatmap in Figure 3C highlights the distinct transcriptional profiles between wildtype and knockout cells at baseline.

Of interest is the expression of CXCL8 and IL6 after TNFα stimulation. In RPS4Y1 KOs, the expression of *CXCL8* is downregulated (Figure 3D). This correlates with the observed suppression of CXCL8 protein production in Figure 2F. Conversely, *IL6* expression is consistent between wildtype and KO cells (Figure 3E), in contrast to the increased production of IL6 protein reported in Figure 2G. RPS4Y1 is a ribosomal protein that contributes to mRNA translation into protein. Our results indicate that in the absence of RPS4Y1, translation of IL6 mRNA into protein might preferentially increase in response to proinflammatory stimuli.

### 2.4. RPS4Y1 Regulates Specific Inflammatory and Migration-Related Pathways

**Figure 4:**
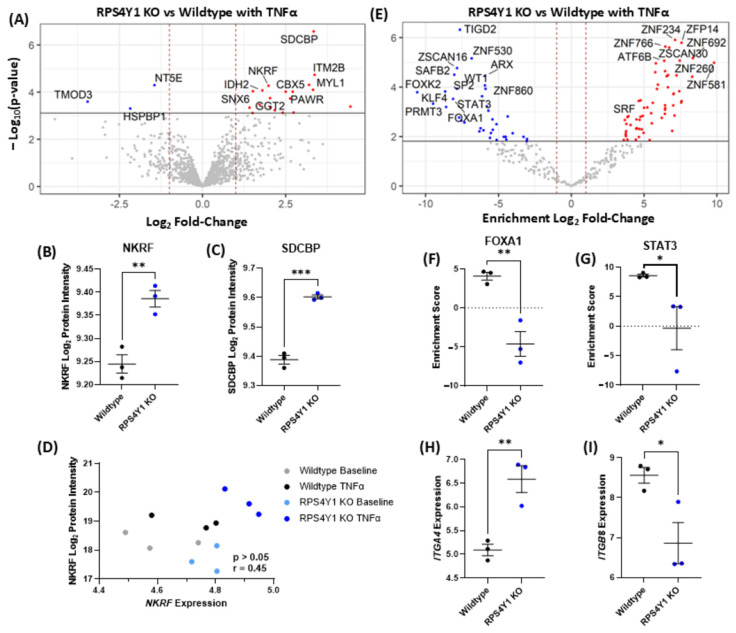
Differential protein abundance and transcription factor enrichment analysis after TNFα stimulation of wildtype and RPS4Y1 KO cell lines. (**A**) Volcano plot of log_10_(*p*-value) against log_2_fold protein abundance change after 24 h TNFα-stimulation (10 ng/mL). Log_2_protein intensity determined by LC-MS/MS for NKRF (**B**) and SDCBP (**C**). (**D**) pQTL linear regression analysis of NKRF log_2_protein intensity against *NKRF* log_2_counts per million (CPM) gene expression in wildtype and RPS4Y1 KO cells. (**E**) Volcano plot of −log_10_(*p*-value) against log_2_ fold-change in transcription factor enrichment score (ARACNE database). For both volcano plots (**A**,**E**), horizontal black lines represented a false discovery rate < 0.05, and dotted red vertical lines indicate log_2_fold-change > |1.0|. Red dots indicate increased production (**A**) or positive enrichment (**E**), and blue dots indicate reduced production (**A**) or negative enrichment (**E**) in RPS4Y1 KOs. (**F**,**G**) transcription factor enrichment score for FOXA1 and STAT3. Log_2_CPM for *ITGA4* (**H**) and *ITGB8* (**I**) in wildtype and RPS4Y1 KOs. (**B**,**C**,**F**–**I**) Data presented as the mean ± SEM and analysed by unpaired parametric *t*-test. (**D**) Analysed by a Spearman correlation with ‘r’ representing the correlation coefficient. Statistical significance indicated by * *p* < 0.05, ** *p* < 0.01 and *** *p* < 0.001. n = 3 for all analyses.

To explore the pathways contributing to *CXCL8* expression and cell adhesion, we analysed the effect of RPS4Y1 KOs on global protein abundances using LC-MS/MS. This analysis revealed that with 24 h TNFα stimulation, three proteins were detected to have reduced abundance in KO cells, with 20 individual proteins increased when RPS4Y1 is knocked out (FDR < 0.05, log_2_FC > 1.0, Figure 4A). NF-κB-repressing factor (NKRF) and syntenin-1 (SDCBP) were essential proteins increased in abundance within RPS4Y1 KOs (Figure 4B,C). Higher levels of NKRF are linked to reduced CXCL8 production [27]. Of note, NKRF gene expression does not correlate with protein production (Figure 4D). The absence of RPS4Y1 may enable more efficient translation of NKRF mRNA into protein, subsequently contributing to the suppression of CXCL8.

To further investigate the contribution of RPS4Y1 to the regulation of gene expression, we completed a transcription factor enrichment analysis within the RNA-sequencing dataset (Figure 4E). This identified multiple differentially activated transcription factor-related pathways. Notably, FOXA1 (Figure 4F) and STAT3 (Figure 4G) were negatively enriched in RPS4Y1 KO cells. As such, genes regulated by these genes demonstrate an overall reduction compared to wildtype cells. Studies have shown that FOXA1 and STAT3 activation significantly increase CXCL8 secretion, and IL8 mRNA levels are reduced when both factors are knocked down. Therefore, reduced activation of the FOXA1 and STAT3 pathways may be driving the suppression of CXCL8 production in combination with increased levels of NKRF.

SDCBP is increased in abundance at a protein level (Figure 4C), with overproduction of this protein linked to the promotion of cell migration and invasion [28]. Pathway analysis using g: Profiler revealed that cell migration and motility are enriched and activated in RPS4Y1 KOs, where integrins mediate increased adhesion in RPS4Y1 KOs (Table 1). In support, we observe increased expression of integrin subunit α4 (*ITGA4*) and decreased expression of integrin subunit β8 (*ITGB8*) in RPS4Y1 KOs (Figure 4H,I). The specific relative abundance of these integrin molecules has been linked with increased adhesion to fibronectin and more significant cell migration.

### 2.5. RPS4Y1 Mediates the Expression and Translation of Specific Extracellular Matrix Proteins

**Figure 5:**
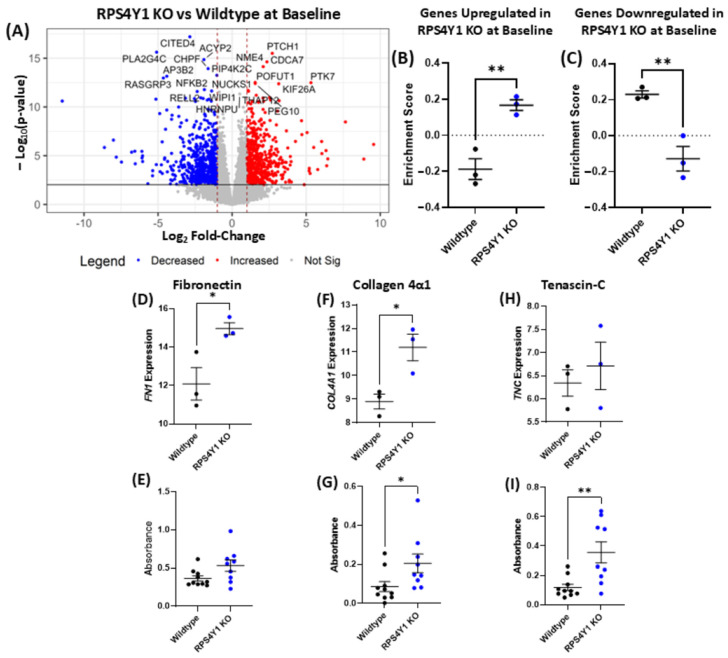
Comparison of gene expression and protein abundance between wildtype and RPS4Y1 KO cell lines. (**A**) Volcano plot of −log_10_(*p*-value) against log_2_fold-change of differentially expressed genes (DEGs) between wildtype and RPS4Y1 KOs after 48 h incubation at 37 °C/5% CO_2_. Horizontal black lines represented a false discovery rate < 0.05, dotted red vertical lines indicate log_2_fold-change > |1.0|. Red dots indicate upregulation, blue dots represent downregulation in RPS4Y1 KOs, and grey dots represent non-significant genes; n = 3. GSVA of up (**B**) or down (**C**) DEGs in RPS4Y1 KOs tracked in protein abundance dataset; n = 3. Log_2_ counts per million (CPM) of *FN1* (**D**), *COL4A1* (**F**) and *TNC* (**H**); n = 3. Fibronectin (**E**), collagen 4α1 (**G**) and tenascin-C (**I**) were measured by ECM ELISA. Higher levels of absorbance (y-axis) indicate a higher level of protein abundance; n = 6–9. Data are presented as the mean ± SEM and analysed by unpaired parametric t-test. Statistical significance is represented by * *p* < 0.05 and ** *p* < 0.01.

The extracellular matrix (ECM) and its integral proteins are important factors that mediate cell proliferation, migration and airway remodelling in asthma. Changes to these proteins are prominent in asthma and contribute to a worse disease prognosis. As such, we investigated how asthma-associated ECM proteins fibronectin (FN1), tenascin-C (TNC) and collagen 4α1 (COL4A1) are altered at a transcriptional and protein level in the absence of RPS4Y1. Differential gene expression analysis revealed 1315 differentially expressed genes between RPS4Y1 KOs and wildtype cells at baseline (Figure 5A). To explore the correlation between changes in gene expression and protein production, GSVA was used to analyse whether differentially expressed genes have similar changes in protein abundance in the LC–MS/MS proteomics dataset. This analysis indicated that genes upregulated or downregulated in RPS4Y1 KOs at baselines were similarly altered at a protein level (Figure 5B,C).

Amongst the differentially expressed genes identified in Figure 5A, fibronectin and collagen 4α1 gene expressions are significantly increased (Figure 5D,F). This increase in mRNA expression is reflected at a protein level measured by ECM ELISA. Fibronectin indicates a trend towards increased production from RPS4Y1 KO cells (Figure 5E), whilst collagen 4α1 production from KO cells is approximately double what wildtype cells produce (Figure 5G). Conversely, tenascin-C indicates similar expression between wildtype and RPS4Y1 KO cell lines (Figure 5H) yet demonstrates significantly increased production at a protein level when RPS4Y1 is knocked out (Figure 5I). TNC is closely implicated in promoting increased cell adhesion and migration in asthma. Therefore, we identify that RPS4Y1 has a complex and dynamic role in regulating the transcription and translation of extracellular matrix proteins. RPS4Y1 appears to mediate cell migration and attachment by producing tenascin-C and interacting with crucial integrin receptors identified in Figure 5H,I.

### 2.6. Genes Altered in RPS4Y1 Knockouts Associate with Asthma Severity in Males

**Figure 6:**
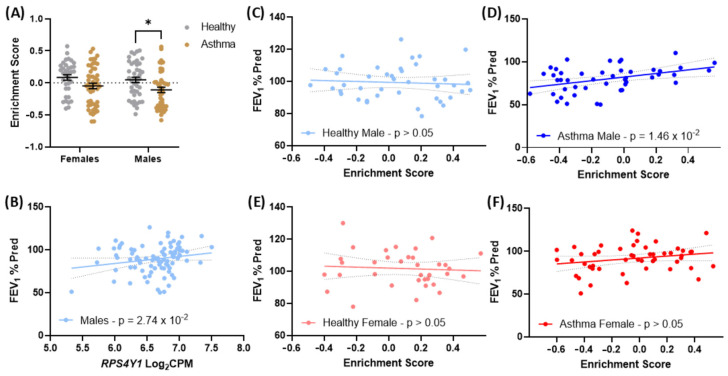
*RPS4Y1* expression and GSVA of upregulated genes in RPS4Y1 KO cells at baseline in healthy and asthmatic patients. (**A**) A subset of the top 50 significant upregulated genes in RPS4Y1 KO cells compared to wildtype cells w analysed for how they change in patients with and without asthma. Grey represents healthy patients, and gold represents asthmatic patients. Data are presented as the mean ± SEM and analysed by two-way ANOVA with Sidak’s correction for multiple comparison testing. Statistical significance is represented by * *p* < 0.05 (**B**) Correlation of log_2_ counts per million (CPM) expression of RPS4Y1 against FEV_1_% predicted scores in males (n = 88). GSVA enrichment scores generated in (**A**) were correlated with the FEV_1_% predicted scores for each patient and stratified by sex and disease. (**C**) healthy males; n = 42, (**D**) asthmatic males; n = 46, (**E**) healthy females; n = 35 and (**F**) asthmatic females; n = 50. Data are presented with 95% confidence intervals indicated by the dotted grey lines. Statistical significance by linear regression analysis with statistical significance determined at a *p*-value < 0.05 with correction for smoking pack years.

RPS4Y1 has broad regulatory functions affecting inflammatory and fibrotic factors. As such, we conducted GSVA using genes upregulated in RPS4Y1 KOs and tracked their expression in bronchial biopsies from healthy patients and patients with asthma. We observe that genes upregulated in RPS4Y1 KOs are significantly decreased in asthmatic patients (Figure 6A). Further, using a linear regression model, *RPS4Y1* expression positively correlates with FEV_1_% predicted in healthy and asthmatic males (Figure 6B). That is, increased expression of *RPS4Y1* correlates with better lung function outcomes. We followed up on these data by exploring how genes upregulated in RPS4Y1 KOs contribute to FEV_1_% predicted scores using linear regression analysis. We observed that genes upregulated when RPS4Y1 is knocked out do not correlate with any change in FEV_1_% predicted in healthy male and female patients (Figure 6C,E) or female asthma patients (Figure 6F). However, we report a significant positive correlation in males with asthma (Figure 6D). No significant association between downregulated genes in RPS4Y1 KOs with asthma or FEV_1_% was predicted for either males or females (Supplementary Figure S4). These results indicate that genes usually suppressed by the function of RPS4Y1 are associated with worse lung function outcomes and asthma severity. When these genes have higher levels of expression, patients demonstrate better lung function measurements. As females do not express *RPS4Y1*, we do not expect a significant effect to be observed. However, as *RPS4X* expression does not change in asthma (Figure 1F), reduced expression of *RPS4Y1* has functional consequences as recognised through FEV_1_% predicted measurement in asthma.

## 3. Discussion

This study shows that *RPS4Y1* expression is cell-specific and different to its X-linked counterpart, *RPS4X,* in asthma. Further, *RPS4Y1* expression was lower in male asthma patients than in male controls, while RPS4X expression did not change in asthma for either sex. We show that RPS4Y1 is involved in regulating proinflammatory cytokines CXCL8 and IL6. The combination of transcriptomic and proteomic analyses allowed us to explore potential pathways by which RPS4Y1 regulates CXCL8 and IL6 production. As such, we uncover that in contrast to CXCL8, whose mRNA expression is altered, IL6 protein translation is increased upon TNFα stimulation, whilst mRNA expression remains unchanged in the wildtype compared to RPS4Y1 KOs. The apparent selectivity of RPS4Y1 to regulate the translation of protein extends to extracellular matrix proteins that are dysregulated in asthma [29,30,31], which we hypothesise promotes altered fibrotic processes of cell adhesion and migration. Finally, we generate an RPS4Y1-specific gene signature that only indicates an association with disease severity in asthmatic males. Altogether, these data highlight that the RPS4Y1 function is altered in asthma and may contribute to unique gene expression profiles between males and females.

Some studies have identified an association between RPS4Y1 expression and asthma [22] through various immune cells [23,32]. Our study shows that RPS4Y1 affects the production of CXCL8 and IL6, which subsequently mediate the infiltration and activation of immune cells such as neutrophils and macrophages, driving the proinflammatory response in the airways [33]. A similar finding was reported by Chen et al. [34], where RPS4Y1 knockdown reduced CXCL8 and IL6 production in endothelial cells with high glucose stimulation. In contrast, we show in RPS4Y1 KOs, CXCL8 production is suppressed whilst IL6 is increased. This indicates that the contribution of RPS4Y1 may be cell and stimulus-specific.

*SPDEF* gene expression is significantly reduced in RPS4Y1 KOs at both baseline and after TNFα stimulation, with this change reported to be greater than that in wildtype cells. *SPDEF* is well-known to be increased in airway epithelial cells of asthma patients [35,36], inducing goblet cell metaplasia and increasing mucous production [37,38]. Both mucous hypersecretion and increased goblet cell density are associated with asthma severity via increasing airway wall thickness [39]. McKay and Hogg posit that any alteration in airway wall structure may have worse effects on airflow in male children than in females [39]. Therefore, dysregulation of *SPDEF* expression by RPS4Y1 may predispose male children to asthma development.

Notably, the immunoregulatory effect at a protein level was not completely reflected at a transcriptional level. CXCL8 mRNA production was reduced, like protein levels, which is driven by increased production of NKRF and activation of the FOXA1 transcription factor pathway. Increased NKRF production has been shown to cause suppression of CXCL8 in A549 cells [27]. Comparatively, FOXA1 knockdown was associated with reduced CXCL8 mRNA levels in two cell models and recovered upon induction of FOXA1 [40]. As such, the combination of these two factors being dysregulated in RPS4Y1 KOs results in decreased suppression of CXCL8. Of significant interest, the expression of *IL6* in KO cells was equal to what was measured in wildtype cells. This indicates that in the absence of RPS4Y1, *IL6* mRNA is preferentially translated to protein. This mechanism is highly complex, with increased IL6 protein being produced due to the lack of RPS4Y1 subunit, enabling greater affinity with the ribosomal, resulting in increased translation, or RPS4Y1 may function through extra-ribosomal means that are poorly defined.

Nonetheless, despite as little as 10% of ribosomes containing RPS4Y1 [9], there is a distinct, specific, and novel regulatory function of RPS4Y1 in IL6 production. Females do not express RPS4Y1, with further studies required to determine whether RPS4X carries similar regulatory functions. However, we hypothesise this would not be the case due to the high expression level of RPS4X in males compared to RPS4Y1. This indicates a significant function for RPS4X supported by most ribosomes containing the X-linked version. Therefore, it is highly likely that RPS4Y1 carries a distinct immunoregulatory function. The latter may contribute to immunological differences observed been males and females with asthma.

Pathway analysis revealed that RPS4Y1 also functions to regulate cell adhesion and migration. RPS4Y1 knockdown has been associated with increased cell invasion [41]. Integrin-mediated cell adhesion is identified to be enriched in RPS4Y1 KO cells, driven by increased expression of *ITGA4* and decreased expression of *ITGB8*. The relative expression of both integrin subunits is associated with increased adhesion to fibronectin and the promotion of cell migration. The ECM is a highly complex scaffold that is carefully regulated in the lungs to enable a robust response in the airways to external stimuli. As such, any dysregulation in the production of ECM proteins may contribute to airway remodelling and worse lung function outcomes in asthma patients. We identify a complex regulatory function of RPS4Y1 for three pertinent proteins in the context of asthma: fibronectin, tenascin-C (TNC) and collagen 4α1. Although RPS4Y1 KOs have increased transcription of fibronectin and collagen 4α1, this does not increase protein production levels.

In contrast, *TNC* gene expression is similar between wildtype and RPS4Y1 KOs, yet KO cells demonstrate significantly increased production of TNC protein. This is unique, as TNC is usually reduced in normal adult tissues [42]; yet, TNC is increased in the airways of asthmatics [43]. TNC is known to directly contribute to processes of cell motility and migration [44], which may explain the increased rate of wound closure observed in RPS4Y1 KOs compared to wildtype cells. These results reinforce the complex function of RPS4Y1 in promoting and impeding the translation of ECM protein. Nonetheless, these data highlight a strong relationship between RPS4Y1 and ECM proteins that are prominent in asthma, indicating a role for RPS4Y1 in asthma progression.

The functional contribution of RPS4Y1 to the clinical outcomes of patients with asthma is confirmed through our correlation of GSVA enrichment scores and lung function outcomes. Genes upregulated in RPS4Y1 KOs cells at baseline demonstrated a positive correlation with the severity of airflow obstruction as reflected by FEV_1_% predicted only in male patients with asthma. Importantly, this finding reinforces the notion that disease pathways related to asthma severity and clinical phenotype are distinct between males and females and require deeper investigation.

A multi-omics approach incorporating RNA-sequencing, proteomics and functional studies is a significant strength of this investigation. This design allowed the identification of complex regulatory contributions of RPS4Y1 impacting gene transcription, protein translation, or potentially post-translational mechanisms. Furthermore, we have identified novel functional effects of RPS4Y1 that directly relate to the hallmark features of asthma–inflammation and fibrosis. We have also identified potential pathways by which RPS4Y1 may mediate these outcomes, but further, more specific and carefully designed studies are required to validate and holistically explore these mechanisms.

Our findings provide a strong foundation for future studies to further elucidate and validate the role of RPS4Y1 in the context of asthma. The current work’s translational application is limited, where follow-up experiments utilising primary human asthmatic epithelial cells would provide a disease-specific understanding of the functional role of RPS4Y1. We provide some indication of a contribution to lung function outcomes via our GSVA analyses.

We identify broad expression of both RPS4X and RPS4Y1 leveraging single-cell RNA-sequencing data (Figure 1G–J). We used this analysis to identify potential cell-type-specific expression of *RPS4Y1* or *RPS4X*. However, it is important to note that this patient population included a broad age range (10–76 years). This captures pubescent and pre-/post-menopausal individuals (i.e., patients with a complex range of sex hormone levels). There is potential for these different hormone levels to affect gene expression on a cell-specific level, which is not captured by our non-age-stratified analysis. Although beyond the scope of the current work, it is pertinent to elucidate age-dependent gene expression, considering the complex network of sex hormones and sex chromosomes on gene expression [45,46].

Further, the role of RPS4Y1 in regulating cellular processes may be cell-specific. In particular, we highlight the dysregulation of well-known ECM factors (fibronectin, collagen 4α1, tenascin-C). Although epithelial cells are known regulators of the ECM, they are not the only cells contributing to remodelling in asthma. These results show a potentially larger pan-cellular role for RPS4Y1 in airway remodelling. As such, it is pertinent to explore its various immunomodulatory or fibrosis in relevant cell types (i.e., fibroblasts). Understanding cell-specific functions of pathologically relevant genes provides more avenues to target different facets of disease progression and improve patient outcomes.

Due to the vital role of RPS4X, evidenced by the analysis of the DepMap database and supported by multiple studies [4,9,11], it was not possible to generate an RPS4X KO cell line. Cases of Turner syndrome, where one copy of the X chromosome is missing in females, highlight that only one copy of RPS4X is necessary for survival, although various medical and developmental problems exist [5]. Therefore, reduced overall expression of *RPS4X/Y1* in males (Figure 1C,D) indicates that males may carry a greater health susceptibility to disease development. This increased predisposition could manifest in childhood before sex hormone production and activity are increased. *RPS4Y1* expression correlates with FEV_1_% predicted scores, indicating a potential contribution to physiological changes linked to disease manifestation.

We have identified that RPS4Y1 regulates hallmark asthma-relevant pathological processes of inflammation, cell adhesion and migration, and mediation of the extracellular matrix. These results implicate RPS4Y1 in the divergent development and progression of asthma between males and females. The use of an inducible RPS4X knockout model or small interfering RNAs may allow the investigation of the function of RPS4X, enabling a comparison to RPS4Y1. This work would support a deeper understanding of ribosomal mechanisms and specific differences between RPS4Y1 and RPS4X. As such, these deeper investigations of the identified pathways and mechanisms will open the opportunity for identifying new and more effective clinical interventions to improve patient outcomes.

## 4. Materials and Methods

### 4.1. Analysis of RPS4X and RPS4Y1 Gene Expression Patterns

The publicly available single-cell RNA-sequencing database ‘Lung Cell Atlas’ (https://asthma.cellgeni.sanger.ac.uk/; accessed on 28 May 2020) was accessed to analyse the gene expression of *RPS4X* and *RPS4Y1* in different epithelial cell types in non-asthmatic (n = 11) and asthmatic patients (n = 9) [47]. A single-cell dataset obtained from the ‘Human Lung Cell Atlas’ (n = 107) [48] was used and stratified by sex for the visualisation of *RPS4X* and *RPS4Y1* expression by uniform manifold approximation and projection (UMAP). These analyses enabled visualisation of gene expression profiles in cell sub-populations as well as in healthy and disease contexts. A detailed description of data processing, analysis and patient demographics for the respective datasets is available in Supplementary Tables S1 and S2.

Two independent cohort studies of asthmatic patients analysing bronchial biopsies (Indurian study) [49] and nasal brushings (OLiVIA study) [50] were used to assess RPS4X and RPS4Y1 expression in male and female participants. A detailed description of each study is in the Supplementary File in Supplementary Tables S3 and S4.

### 4.2. Analysis of RPS4X and RPS4Y1 Essentiality for Cell Survival

The Cancer Dependency Map (DepMap; https://depmap.org/portal/interactive/; accessed on 18 June 2022) online tool was used to investigate the effect of knocking out *RPS4X* and *RPS4Y1* on cell survival. The CRISPR loss-of-function screen was evaluated using the Chronos algorithm [51] to infer the effect of gene knockout on cell fitness as provided by DepMap. The ‘gene essentiality score’ for *RPS4X* and *RPS4Y1* was filtered for lung-derived cell lines. A score less than minus one indicates that the gene is vital for cell survival, meaning the cell will not survive if the gene is knocked out. A score greater than 0.5 indicates the gene contributes to cell proliferation.

### 4.3. Generation of CRISPR Cas9 Genome Deletion Cell Lines

**Figure 7:**
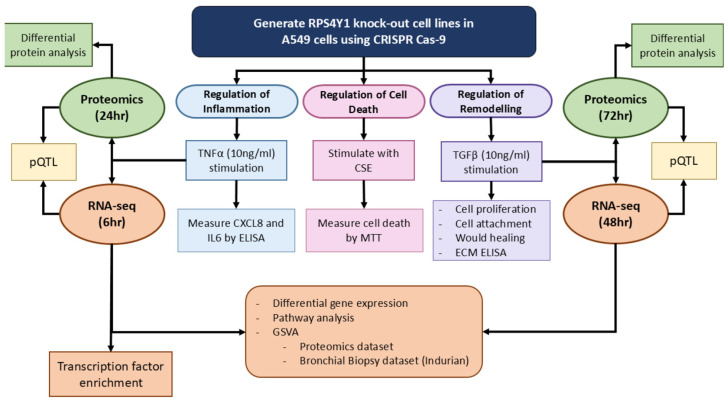
Schematic representation of the study design, techniques and analyses used.

A schematic representation of the overall study design and analyses is included in Figure 7. The online web tool Benchling (version 2020) was used to design guide RNA (gRNA) sequences. gRNA probes were designed to target a common exon present for all known splice variants for *RPS4Y1*. Guide sequences contained the protospacer adjacent motif (PAM) sequence NGG. The appropriate guide sequence was selected based on a high on-target specificity score and 20 base pairs. gRNA pairs were incorporated into the pX458 CRISPR-Cas9 plasmid (Addgene, Watertown, MA, USA), and after positive selection by ampicillin, plasmids were amplified in *Escherichia coli* DH5α heat-competent bacteria (NEB). After expansion, plasmids were isolated and purified using the Qiagen Plasmid Mini Kit (Qiagen) following the manufacturer’s instructions. Purified plasmid constructs were transfected into human lung adenocarcinoma (A549) cells (ATCC) using lipofectamine 3000 transfection reagent (ThermoFisher, Waltham, MA, USA) following the manufacturer’s instructions. Transfected cells were single-cell sorted and selected based on green fluorescent protein expression (GFP+). Single cells were expanded following sorting and clonally expanded. Knockout (KO) status was validated by genome sequencing. Sequencing data was generated by AGRF, with results assessed using the ICE CRISPR Analysis tool (https://ice.synthego.com/#/; accessed on 20 August 2019) and Benchling. A clonally expanded ‘reagent control’ cell line, which underwent all the above processes minus transfection, is used throughout this study as a ‘wildtype’ control cell line.

### 4.4. Cell Culture and Treatments

All generated cell lines were maintained in DMEM supplemented with 10% FBS (growth medium). For all experiments, cells were seeded at a density of 25,000 cells/mL in 12-well cell culture plates unless otherwise stated. Once 80% confluency was reached, wildtype and knockout A549 alveolar epithelial cells were washed twice with HANKS balanced salt solution (HBSS) (Sigma-Aldrich, Burlington, MA, USA) and quiesced in DMEM supplemented with 0.1% bovine serum albumin (BSA) (Sigma-Aldrich, Burlington, MA, USA) for 18 h. For inflammatory studies, cells were stimulated with TNFα (10 ng/mL) for either 6 or 24 h. For the study of fibrotic changes cells were stimulated with TGF-β (10 ng/mL) for 48 or 72 h.

### 4.5. Western Blotting

RPS4Y1 and GAPDH protein levels were detected by western blotting of knockout and wildtype cell lines, as previously described [52]. Briefly, whole-cell protein lysates were collected in protease inhibitors containing lysis buffer (#539132, Sigma-Aldrich, Burlington, MA, USA). An amount of 40 µg of total protein was electrophoresed and transferred to a polyvinyl difluoride membrane (#IPVH00010, Merck-Millipore, Darmstadt, Germany) and blocked for one hour at room temperature using skim milk. Membranes were then incubated either with a 1:1000 dilution of rabbit anti-human RPS4Y1 (#17296-1-AP, ProteinTec, Rosemont, IL, USA) or a 1:5000 dilution of mouse anti-human GAPDH (#MAB374, Merck-Millipore, Darmstadt, Germany) primary antibodies overnight at 4 °C. Membranes were washed and then incubated with the secondary horseradish peroxidase linked antibody; 1:10,000 dilution of rabbit anti-mouse antibody (#AP160P, Merck-Millipore, Darmstadt, Germany) or 1:2000 dilution of goat anti-rabbit antibody (#P0448, Dako, Dresden, Germany). Chemiluminescent substrates (PerkinElmer, Waltham, MA, USA) were used to visualise protein bands. GAPDH bands functioned as a loading control.

### 4.6. Measurement of CXCL8 and IL6 Protein Secretion

Cell-free supernatants were collected 24 h post-stimulation, and the concentration of CXCL8 and IL6 was quantified by an enzyme-linked immunosorbent assay (ELISA). ELISA was completed as previously described by Rutting et al. [53].

### 4.7. RNA-Sequencing

Whole-cell RNA extracts were collected from wild type and RPS4Y1 KO cell lines using Isolate II RNA isolation kits (Bioline, London, UK), following the manufacturer’s protocol. Sample quality was assessed using a Nanodrop. Only samples with an A260/280 ratio between 1.8 and 2.1 and RIN greater than 8 were sent to the Ramaciotti Centre for Genomics to conduct RNA sequencing. Samples were prepared by Illumina Stranded mRNA prep ligation, where RNA integrity was also confirmed to be greater than 8. Single-end sequencing was completed by polyA tail pulldown on a NovaSeq6000 S1 flowcell. RNA-sequencing results were aligned to GENCODE v38 with reads counted using a STAR aligner. Differential gene expression analysis was completed using the *DESeq2* package in ‘R’. Data was normalised by variance stabilizing transformation using the *vst* function from *DESeq2* for all KO RNA-seq associated data.

### 4.8. Transcription Factor Enrichment Analysis

ARACNE (http://wiki.c2b2.columbia.edu/califanolab/index.php/Software/ARACNE; accessed on 6 June 2022) was used to identify transcription factors using a list of human transcription factors (n = 1639) downloaded from the Human Transcription Factor database (http://humantfs.ccbr.utoronto.ca/; accessed on 6 June 2022). A total of 100 bootstrap rounds were applied, and all inferred networks were merged to obtain a consensus network. Viper was then applied to infer the protein activity of the identified transcription factor [54]. Transcription factors that had direct ARACNe-predicted T (regulon) statistically enriched in the gene expression signature were selected as the most representative transcription factors.

### 4.9. Differential Gene Expression Analysis

Differential gene expression (DGE) analysis was completed using negative binomial generalised linear models with the R package ‘*DESeq2*’ (version 4.2.0). Standard DGE analysis compared the differences in gene expression between each cell line under the same treatment conditions. Interaction DGE analysis compared the change in gene expression of one cell line when stimulated and at baseline to the change in gene expression of another cell line when stimulated and at baseline. Interaction DGE analysis reveals genes uniquely regulated after treatment in each cell line. Correction for multiple testing was applied by controlling the false discovery rate (FDR) by the Benjamini–Hochberg (BH) methodology. Significant differentially expressed genes were determined at an FDR < 0.05 and a log_2_fold-change > |1.0|.

### 4.10. Analysis of Biological Pathways Enriched in Knockout Cell Lines

Pathway analysis was completed to identify the biological pathways that involve the significantly differentially expressed genes in knockout cell lines compared to wildtype cells. The g: Profiler web-based tool was used to complete the analysis, using a subset of at most the top 50 significant genes (FDR < 0.05 and FC > |1.0|).

### 4.11. Wound Healing Assay

Wound healing assays were conducted once cells had reached confluency. A scratch was created using a P200 pipette tip through the centre of a cell monolayer in a 12-well plate. Wells were washed with HBSS twice after the scratch was made. DMEM growth medium was replaced, and cells were incubated at 37 °C/5% CO_2_. An image of the wound was taken at 0 h (baseline) on a Nikon Eclipse Ti microscope at 4× magnification. Subsequent images were captured at the same location in the well at 24, 48 and 72 h. Wound sizes were analysed using the ImageJ (v1.53) software plug-in “Wound Healing Size” [55].

### 4.12. Proliferation Assay

Proliferation was investigated by seeding 400,000 cells in a T75 cm^2^ flask. The flasks were incubated at 37° C/5% CO_2_ for four days in growth media. After incubation, cells were harvested using trypsin and counted using a haemocytometer. The number of cells was then compared to the starting seeding concentration.

### 4.13. Cell Adhesion to Fibronectin

To measure the fibronectin-mediated adhesion of cells, an adapted protocol from Humphries [56], was used. Briefly, 96-well plates were coated with fibronectin at 5 µg/cm^2^ (ThermoFisher, Waltham, MA, USA) at room temperature, with a set of wells left uncoated as controls. Cells were seeded at a density of 1 × 10^5^ cells/mL in DMEM supplemented with 1% (*v*/*v*) FBS and incubated for one hour at 37° C/5% CO_2_. Cell attachment was detected using crystal violet staining of adhered cells after washing to remove unattached cells. The complete methodology is in the Supplementary File. The relative number of attached cells was determined by measuring absorbance at 570 nm (SpectraMax iD3, Molecular Devices, Fremont, CA, USA).

### 4.14. Measurement of ECM Protein

To assess changes in extracellular matrix (ECM) protein deposition between the knockout cell lines, an ECM ELISA was performed as previously described [57]. Cells were grown in a 96-well plate and serum-starved for 18 h before stimulation with TGF-β (10 ng/mL) for 72 h. Cells were removed using NH_4_OH at 37 °C for 15 min. The cell-free ECM plates were analysed for the deposition of ECM proteins according to the previously described method [57]. Primary antibodies used were Fibronectin (#MAB1935, clone 868A11, Sigma-Aldrich, Burlington, MA, USA), tenascin-C (#T2551, clone BC-24, Merck-Millipore, Darmstadt, Germany) and collagen IV (#C1926, clone COL-94, Sigma-Aldrich, Burlington, MA, USA). Rabbit anti-mouse monoclonal HRP-linked (#7076S, Cell Signalling, Danvers, MA, USA) was used as a secondary antibody. IgGκ isotype control (#557273, BD Pharminogen, San Diego, CA, USA) was used at the same concentration as the primary antibodies.

### 4.15. Cigarette Smoke Extract (CSE) Generation and Analysis of Cell Death Response

Marlboro Red standard cigarettes (Philip Morris, Stamford, CT, USA) were used to prepare CSE using a method described previously [58]. However, in this study, smoke from two Marlboro cigarettes was bubbled through 25 mL of unsupplemented DMEM at a constant rate, with this solution considered as 100% concentrated CSE. This 100% CSE was freshly generated for each experiment, diluted to the final working concentration, and used within 15 min.

### 4.16. Cell Viability Assay

Cells were seeded in 96-well plates at a density of 2 × 10^4^ cells/mL and were serum-starved before stimulation with CSE dilutions of 50%, 75%, and 100%. The mitochondrial activity of the living cells was tested using a Thiazolyl blue tetrazolium bromide (MTT) assay (Sigma-Aldrich, Burlington, MA, USA). After stimulation for 24 h, MTT dye was added to the supernatant, with plates incubated at 37 °C/5% CO_2_ for 4 h. MTT dye was solubilised with dimethyl sulfoxide (DMSO). MTT dye was measured using a spectrophotometer (SpectraMax iD3, Molecular Devices, Fremont, CA, USA) at 570 nm and background at 630 nm.

### 4.17. LC-MS/MS Proteomics Analysis

Whole-cell lysates were collected using 1% sodium deoxycholate in 100 mM HEPES pH 8.5 and digested with trypsin at 1:100 (*v*/*v*). Digested samples were cleaned and processed using an adapted solid-phase extraction method [59]. Detailed methods are in the supplementary file. Briefly, protein samples were analysed following a data-dependent MS/MS experiment and searched using MaxQuant (v2.2.0.0) analysis software against the UniProt human reference proteome database (downloaded 1 March 2021) with label-free quantification (LFQ) specified. The search results were input into LFQ-Analyst [60] for differential protein abundance analysis with least-squares linear modelling, and correction for multiple testing was applied by controlling the false discovery rate (FDR) using the Benjamini–Hochberg (BH) procedure. Statistical significance was determined at an FDR < 0.05 and log_2_fold-change > |1.0|.

### 4.18. Gene Set Variation Analysis (GSVA)

GSVA investigated sample-wise gene set enrichment scores [61] to understand the transcriptomic changes in one cell line compared to another. This analysis was also conducted using the protein abundance dataset generated by LC/MS-MS. We conducted GSVA on the bronchial biopsies collected from asthmatic and healthy control patients to analyse the association between knockout cell line gene expression and clinical phenotypes.

### 4.19. Protein Quantitative Trait Loci (pQTL) Validation

Protein quantitative trait loci (pQTL) analysis compared gene expression to protein abundance. Gene expression at 6 h was compared to protein abundance after 24 h, whilst gene expression at 48 h was compared to protein abundance after 72 h. pQTL analysis was completed in ‘R’ using ‘*MatrixEQTL*’. The association between gene expression and protein abundance was tested using linear regression with additive ANOVA or gene expression effects. The Benjamini–Hochberg methodology was used to account for multiple comparisons testing. A significant association was determined at an FDR < 0.05.

## 5. Conclusions

We show for the first time that RPS4Y1 regulates the protein translation of specific proteins, namely IL6 and ECM factors. We further identify the regulatory transcription factor pathway altered when RPS4Y1 is knocked out, potentially contributing to suppressed gene expression and protein production of CXCL8. Furthermore, we show that RPS4Y1 mediates the expression of integrin subunits α4 and β8, which are associated with tenascin-C, increasing cell adhesion and migration, which are known to contribute to worse asthma outcomes. We also establish that RPS4Y1 regulates a specific gene signature that is only dysregulated in males with asthma, indicating that the imbalance of RPS4Y1 and RPS4X expression between males and females may contribute to sex differences in asthma. Importantly, this study provides more weight to the growing work investigating sex differences in asthma and other prominent respiratory diseases. As such, future studies can build on the findings of the current work to better explore the mechanisms driving sexual dimorphism in disease.

## Figures and Tables

**Table 1:** g:Profiler pathway analysis of RNA-seq data using significantly up and down-regulated genes in RPS4Y1 KO compared to wildtype at baseline.

Positively Enriched in RPS4Y1 KO at Baseline		Negatively Enriched in RPS4Y1 KO at Baseline	
Pathway	FDR	Pathway	FDR
Regulation of cell migration	4.33 × 10^−7^	Actin binding	1.47 × 10^−2^
Regulation of cell motility	1.05 × 10^−7^	Guanine metabolic process	3.56 × 10^−2^
Regulation of signalling	2.21 × 10^−3^	Myosin complex	4.71 × 10^−2^
Cell adhesion mediated by integrins	2.36 × 10^−3^		

FDR = false discovery rate.

## Data Availability

The RNA sequencing data used in this study are publicly available through the NCBI Gene Expression Omnibus. The datasets analysed throughout the current study are published online or deposited on the NCBI Gene Expression Omnibus (GSE299878). Bronchial biopsy (Indurain) data can be accessed through the European Genome Archive at EGA#337622.

## Supplementary Materials

The following supporting information can be downloaded at https://www.mdpi.com/article/10.3390/ijms26136213/s1 (References [62,63] are cited in the supplementary materials).

## Abbreviations

The following abbreviations are used in this manuscript:

A549	Adenocarcinoma human alveolar basal epithelial cells
AGRF	Australian Genomes Research Facility
ANOVA	Analysis of variance
APS	Ammonium persulfate
ARACNe	Algorithm for the Reconstruction of Gene Regulatory Networks
BH	Benjamini–Hochberg
Col4α1	Collagen 4 alpha chain 1
CPM	Counts per million
CRISPR	Clustered regularly interspaced short palindromic repeats
CSE	Cigarette smoke extract
CXCL8	Chemokine (C-X-C motif) ligand 8
DTT	Dithiothreitol
ECM	Extracellular matrix
FDR	False discovery rate
FEV_1_	Forced expiratory volume in 1 s
FEV_1_% Pred	Percentage of the patient FEV_1_ divided by the average FEV_1_ of the population
FN1	Fibronectin
GFP	Green fluorescent protein
gRNA	guide RNA
GSVA	Gene set variation analysis
IL-1β	Interleukin-1 beta
indel	Insertion or deletion of DNA bases
ITGA4	Integrin subunit alpha 4
ITGB8	Integrin subunit beta 8
LC-MS/MS	Liquid chromatography—tandem mass spectrometry
LFQ	Label-free quantification
MTT	3-4, 5-dimethylthiazol-2-(yl)-,5, diphenyltetrazolium
NH_2_OH	Hydroxylamine
OLiVIA	Effects Of Extra-fine ParticLe HFA-Beclomethasone Versus Coarse Particle Treatment In Smokers and Ex-smokers with Asthma
PAM	Protospacer adjacent motif
PMSF	Phenylmethylsulphonyl fluoride
pQTL	Protein quantitative trait loci
PVDF	Polyvinylidene difluoride
RP	Ribosomal protein
RPS4X	Ribosomal Protein S4 X-linked
RPS4Y1	Ribosomal Protein S4 Y-linked 1
rRNA	Ribosomal RNA
SDB-RPS	Styrene divinylbenzene-reverse phase sulfonated
SDS-PAGE	Sodium dodecyl sulfate-polyacrylamide gel electrophoresis
SPE	Solid phase extraction
STAT3	Signal transducer and activator of transcription 3
TEMED	N, N, N′, N′-tetramethyl ethylenediamine
TGF-β1	Transforming growth factor-beta
TNC	Tenascin C
TNFα	Tumour necrosis factor-alpha
UMAP	Uniform manifold approximation and projection for dimension reduction
X-chr	X chromosome
XCi	X-chromosome inactivation
Y-chr	Y chromosome
