# Supplementary material for: A Novel Regulatory Role for RPS4Y1 in Inflammatory and Fibrotic Processes
Source: Int J Mol Sci. 2025 Jun 27;26(13):6213. doi: 10.3390/ijms26136213 (PMC12249968; doi:10.3390/ijms26136213)
Supplement: Supplementary file 1 [file ijms-26-06213-s001.zip › ijms-3653052-supplementary.pdf]

Supplementary File

Methods

Single-cell sequencing ‘Lung Cell Atlas’

The publicly available single-cell RNA-sequencing database ‘Lung Cell Atlas’ (https://asthma.cellgeni.sanger.ac.uk/) was accessed to analyse the gene expression of *RPS4X* and *RPS4Y* in different epithelial cell types from non-asthmatic and asthmatic patients. Patient samples were collected as part of a previous study by bronchoscopy biopsy. Complete details and methodology are published (Vieira Braga et al. 2019). The cohort inclusion criteria for all patients were: aged between 40 – 65 years of age, < 10 pack years smoking history. The criteria for only the patients with asthma: age of onset < 12 years old, documented history of asthma, use of inhaled corticosteroids with(out) β_2-agonists due to respiratory symptoms, and positive provocation test (PC_{20} methacholine – induction of a 20% decrease in forced expiratory volume in the first second < 8 mg/mL with 2 min protocol). For non-asthmatic controls: absent history of asthma, no use of asthma-related medication, a negative provocation test (PC_{20} > 8 mg/mL, and adenosine 5’-monophosphate > 320 mg/mL with 2 min protocol), no pulmonary obstruction (FEV_1/FVC > 70%) and absence of lung function impairment (FEV_1 > 80% predicted). Patients with asthma stopped inhaled corticosteroid use six weeks before testing and protocols were started. Patient demographics, as used in the current study, are detailed in Table S1.

Table S1: Demographics of patients included in the Lung Cell Atlas dataset.

Characteristic	Control	Asthma
**n, (male / female)**	7M / 4F	7M / 2F
**Age, yr**	56	55
**FEV_1 % Predicted**	115.73 (+/- 13.86)	82.22 (+/- 19.56)
**Pack years**	1.27 (+/- 1.85)	0.00 (+/- 0.00)

FEV_1 = forced expiratory volume after one second. Data are presented as arithmetic mean (+/- SD).

Single-cell sequencing ‘Human Lung Cell Atlas’

A single-cell dataset was obtained from the ‘Human Lung Cell Atlas’ (Sikkema et al. 2022) and was prepared using the ‘*Seurat’* R package per the methodology outlined by Hao et al. (Hao et al. 2021). The resulting, processed dataset was stratified by sex with the expression of *RPS4X* and *RPS4Y1* visualised as a UMAP (uniform manifold approximation and projection for dimension reduction). The UMAP identified cell subpopulations such as epithelial cells, alveolar macrophages and immune cells. This enabled visualisation of which cell types the genes were expressed to provide insight into their expression patterns and function. A summary of the demographics for the patients included in the original data collection by Sikkema et al. (Sikkema et al. 2022) is summarised in Table S2.

Table S2: Demographics of patients included in the single cell sequencing Human Lung Cell Atlas.

**Characteristic**	
**n, (male / female)**	64M / 43F
**Age, range (yr)**	10 - 76
**BMI, (range)**	20 - 49
**Smoking Status, n**	
Never smoker	56
Ex-smoker	17
Current smoker	16
Unannotated	18

BMI: body mass index

Indurian bulk RNA-seq bronchial biopsy dataset

Bronchial biopsies were collected from patients as part of a previous study (Vermeulen et al. 2020). The details of the study design and methodology are previously published (Broekema et al. 2010). All data is publicly available (EGA #337622). This study included persistent asthma subjects with clinical remission of asthma, complete remission of asthma and healthy control patients. We have only included patients with persistent asthma and healthy controls for the current study. The patient demographics are summarised in Table S3.

Table S3: Demographics table for the Indurian bronchial biopsy study patients.

**Characteristic**	**Males**	**Females**
**Total, n (% Asthma)**	88 (52.3%)	85 (58.8%)
**Age, yr**	46.46 (+/- 14.19)	41.29 (+/- 15.42) *
**Pack years**	10.42 (+/- 14.47)	5.27 (10.87) **
**FEV_1, L**	3.72 (+/- 0.91)	3.05 (+/- 0.68) ****
**FEV_1 % Predicted**	89.19 (+/- 16.13)	95.77 (+/- 14.59) **
**Sputum cell counts**<br>Absolute (x10^3)<br>% cell count		
**Neutrophil**	1.5 (+/- 2.33)<br>55.07% (+/- 19.35%)	1.68 (+/- 6.27)<br>53.42% (+/- 23.34%)
**Eosinophil**	0.04 (+/- 0.10)<br>1.82% (+/- 4.44%)	0.02 (+/- 0.09)<br>0.94% (+/- 1.80%)
**Macrophage**	1.05 (+/- 1.13)<br>42.45% (+/- 19.53%)	0.97 (+/- 1.43)<br>44.74% (+/- 22.92%)
**Lymphocyte**	0.02 (+/- 0.03)<br>0.66% (+/- 0.96%)	0.02 (+/- 0.03)<br>0.91% (+/- 1.37%)

*FEV_1 = forced expiratory volume after one second. Data are presented as arithmetic mean (+/- SD). Statistical significance is indicated by *p<0.05, **p<0.01, ****p<0.0001, determined by parametric t-test.*

OLiVIA clinical dataset patients and study design

Study subjects were selected from participants of the 'Effects **O**f Extra-fine ParticLe HFA-Beclomethasone **V**ersus Coarse Particle Treatment **I**n Smokers and Ex-smokers with **A**sthma' (OLiVIA study) (ClinicalTrials.gov #NCT01741285)(Cox et al. 2017). This study was approved by the local medical ethics committee, with written and informed consent received before collection. Patients were included if they were either current- or ex-smokers (smoking cessation ≥ 6 months), aged 18 – 65 years old and had doctor-diagnosed asthma with a smoking history of ≥ 5 pack years. Exclusion criteria comprised treatment with oral steroids, FEV_1 ≤ 1.2 L, an upper respiratory tract infection ≤ 4 weeks before inclusion, and an asthma exacerbation ≤ 6 weeks before inclusion. Nasal brushings were taken from the inferior turbinate of patients that met the inclusion criteria. RNA was isolated from the samples and quality tested before single-end sequencing. RNA sequencing data quality control was performed using R (version 3.4.3) to ensure concordance between reported sex and sex-associated genes. Data were $\log_2$ transformed and normalised. The patient demographics are summarised in Table S4.

Table S4: Clinical summary data for OLiVIA study patients.

	Never-smoker	Ex-smoker	Current-Smoker
**n**	18	30	54
**Male, n (%)**	10 (55.6)	13 (43.3)	26 (48.1)
**Age, yr**	50 (14.6)	50 (11.0)	41 (12.4) ** *$*
**Pack years**	0.00 (0.00)	21.10 (19.12)	20.77 (14.15)
**FEV_1, L**	3.68 (0.83)	2.91 (0.70) **	3.43 (0.84) *$*
**FEV_1 % Predicted**	107.4 (12.29)	90.00 (18.21) ***	95.06 (15.13) *

FEV_1 = Forced expiratory volume in one second (litres). Data are presented as the arithmetic mean +/- standard deviation and analysed using one-way ANOVA with Tukey correction for multiple testing. *$p<0.05$ (vs never smoker), **$p<0.01$ (vs never smoker), ***$p<0.001$ (vs never smoker), *$* $p<0.05$ (current smoker vs ex-smoker).

Cell Adhesion Assay

To measure the fibronectin-mediated adhesion of cells, an adapted protocol from Humphries(Humphries 2000) was used. In brief, fibronectin was diluted in sterile PBS at 16µg/ml with 100µl of the solution added to half a 96-well plate. The plate was incubated at room temperature under sterile conditions for 1 hour. The fibronectin solution was removed, and cells were seeded across both coated and uncoated wells at one million cells per ml. One row of the plate was not seeded to control for the natural background of fibronectin in the well. The plate was agitated by semi-forceful tapping to ensure an even distribution of cells before incubation at 37°C/5% CO_2 for one hour. After the incubation, the plate was again tapped with even force to detach any weakly adhered cells. The media was carefully aspirated, and each well was gently washed with sterile PBS. Adherent cells were fixed with 100µl of 4% paraformaldehyde by incubation at room temperature for 20min. Wells were then washed twice with sterile PBS. Adherent cells were stained with 100µl of 0.1% crystal violet, 200mM MES, pH 6.0 for two hours. After staining, cells were washed three times with water before the crystal violet was solubilised with 10% (v/v) acetic acid. The maximise solubilisation, the plate was agitated for one minute. Absorbance was measured at 570nm. Background absorbance (no cell) was subtracted to analyse attachment from all wells. Fibronectin-coated

absorbance was divided by non-fibronectin-coated wells to determine the fold change in adherence.

LC-MSMS Proteomics Analysis

Whole-cell lysates were collected using 1% sodium deoxycholate in 100mM HEPES pH 8.5 buffer and digested using trypsin (1:100 (v/v) ratio). Digested samples were cleaned and processed using an adapted solid phase extraction method from Rappsilber et al.(Rappsilber et al. 2007) using SDB-RPS media. Processed samples were then run using an Acquity M-class nanoLC system (Waters, USA), 5 µL of the sample was loaded at 15µL/min for 3 minutes onto a nanoEase Symmetry C18 trapping column (180µm x 20mm) before being washed onto a PicoFrit column (75 µmID x 350 mm; New Objective, Woburn, MA) packed with SP-120-1.7-ODS-BIO resin (1.7µm, Osaka Soda Co, Japan) heated to 45C. Peptides were eluted from the column and into the source of a Q Exactive Plus mass spectrometer (Thermo Scientific) using the following program: 5-30% MS buffer B (98% Acetonitrile + 0.2% Formic Acid) over 90 minutes, 30-80% MS buffer B over 3 minutes, 80% MS buffer B for 2 minutes, 80-5% for 3 min. The eluting peptides were ionised at 2400V. A data dependant MS/MS (dd-MS^2) experiment was performed, with a survey scan of 350-1500 Da performed at 70,000 resolution for peptides of charge state 2+ or higher with an AGC target of 3e6 and maximum Injection Time of 50ms. The Top 12 peptides were selected and fragmented in the HCD cell using an isolation window of 1.4 m/z, an AGC target of 1e5 and a maximum injection time of 100ms. Fragments were scanned in the Orbitrap analyser at 17,500 resolution and the product ion fragment masses were measured over a mass range of 120-2000 Da. The mass of the precursor peptide was then excluded for 30 seconds. The output MS/MS data files were searched using MaxQuant analysis software against the Uni Prot human database with label-free quantification (LFQ). Search parameters used are as follows; Fixed modifications: None; variable modifications: oxidation, carbaminomethyl, deamination; Enzyme: semi-trypsin; the number of allowed missed cleavages: 3; peptide mass tolerance: 10 ppm; MS/MS mass tolerance: 0.05 Da. The search results were then inputted into the online proteomics visualisation tool, LFQ-Analyst (https://analyst-suite.monash-proteomics.cloud.edu.au/apps/lfq-analyst//)(Shah et al. 2019). Differential protein analysis was conducted with significant differentially expressed proteins determined at an FDR < 0.05 and a fold-change > |1.0|. Multiple testing correction was adjusted for using the Benjamini-Hochberg methodology.

Results

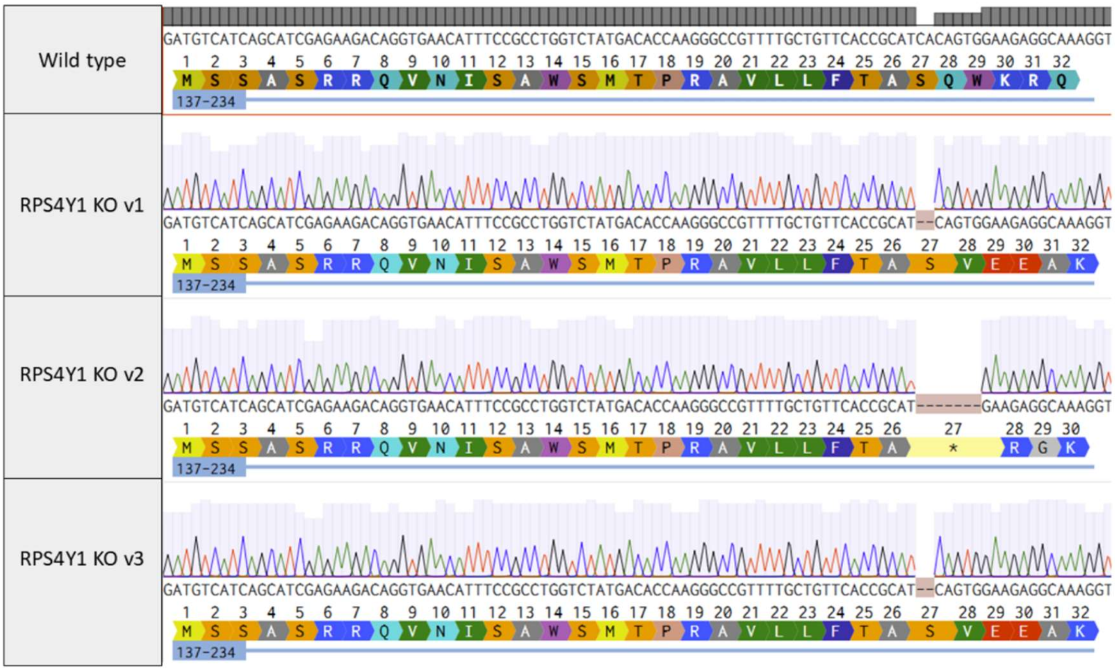

Figure S1: Chromatograms of wildtype and RPS4Y1 knock-out genome sequencing with codon translation to amino acid sequence underneath. Asterisk (*) = stop codon; minus (-) = base deletion.

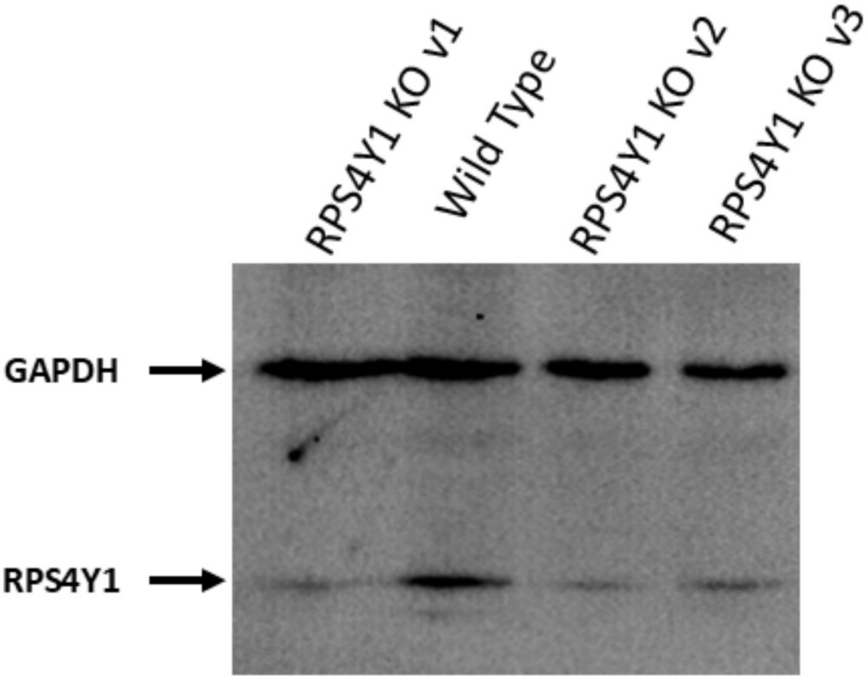

Figure S2: Western blot image of GAPDH (top) and RPS4Y1 (bottom) in wildtype and RPS4Y1 KO cell lines.

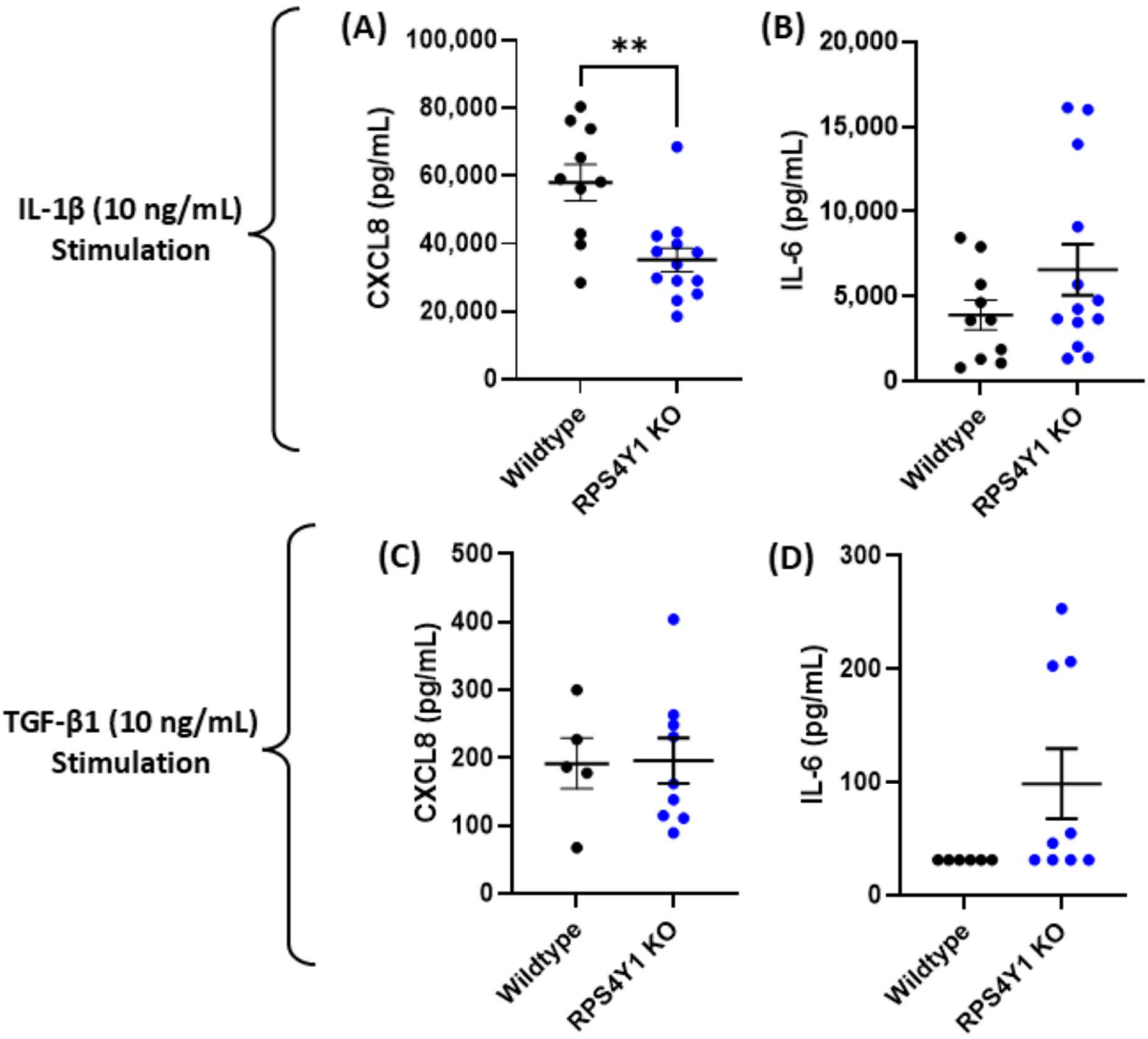

Figure S3: Production of CXCL8 (A & C) and IL6 (B & D) from RPS4Y1 knockout and wildtype cells when stimulated with IL-1β (A & B) and TGF-β1 (C & D). CXCL8 and IL6 were measured after 24-hour IL-1β (10 ng/mL) and TGF-β1 (10 ng/mL) stimulation in cell-free supernatant by ELISA. All data are presented as the arithmetic mean +/- SEM. One-way ANOVA statistical analysis with Tukey's correction for multiple comparisons. Statistical significance is indicated by **$p<0.01$; n = 5 – 13.

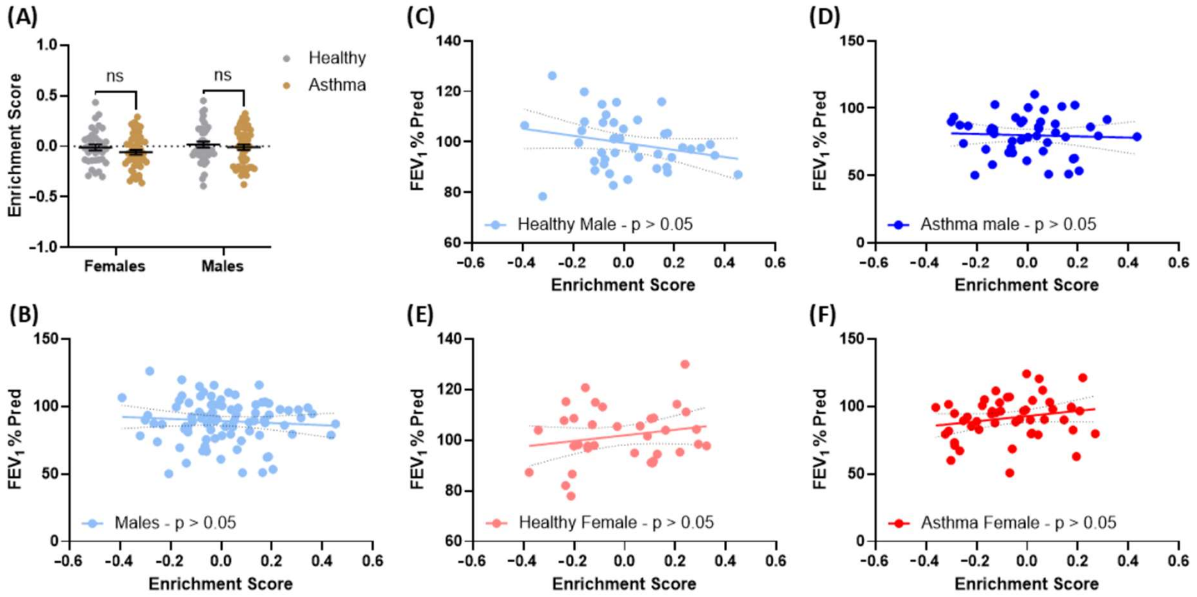

Figure S4: Figure 6: *RPS4Y1* expression and GSVA of downregulates genes in RPS4Y1 KO cells at baseline in healthy and asthmatic patients. (A) A subset of the top 50 significant downregulated genes in RPS4Y1 KO cells compared to wildtype cells were analysed for how they change in patients with and without asthma. Grey represents healthy patients, and gold represents asthmatic patients. Data are presented as the mean +/- SEM and analysed by two-way ANOVA with Sidak's correction for multiple comparison testing. Statistical significance is represented by *p<0.05 (B) Correlation of $\log_2$ counts per million (CPM) expression of RPS4Y1 against FEV_1 % predicted scores in males (n=88). GSVA enrichment scores generated in (A) were correlated with the FEV_1 % predicted scored for each patient and stratified by sex and disease. (C) healthy males; n=42, (D) asthmatic males; n=46, (E) healthy females; n=35 and (F) asthmatic females; n=50. Data are presented with 95% confidence intervals. Statistical significance by linear regression analysis with statistical significance determined at a p-value<0.05 with correction for smoking pack years.

References

Broekema M, Volbeda F, Timens W, Dijkstra A, Lee N, Lee J, Lodewijk M, Postma D, Hylkema M, Ten Hacken N. 2010. Airway eosinophilia in remission and progression of asthma: accumulation with a fast decline of FEV1. *Respiratory medicine* **104**: 1254-1262.

Cox CA, Boudewijn IM, Vroegop SJ, Schokker S, Lexmond AJ, Frijlink HW, Hagedoorn P, Vonk JM, Farenhorst MP, Ten Hacken NH. 2017. Extrafine compared to non-extrafine particle inhaled corticosteroids in smokers and ex-smokers with asthma. *Respiratory Medicine* **130**: 35-42.

Hao Y, Hao S, Andersen-Nissen E, Mauck WM, Zheng S, Butler A, Lee MJ, Wilk AJ, Darby C, Zager M. 2021. Integrated analysis of multimodal single-cell data. *Cell* **184**: 3573-3587. e3529.

Humphries MJ. 2000. Cell adhesion assays. *Extracellular Matrix Protocols*: 279-285.

Rappsilber J, Mann M, Ishihama Y. 2007. Protocol for micro-purification, enrichment, pre-fractionation and storage of peptides for proteomics using StageTips. *Nature protocols* **2**: 1896-1906.

Shah AD, Goode RJ, Huang C, Powell DR, Schittenhelm RB. 2019. LFQ-analyst: an easy-to-use interactive web platform to analyze and visualize label-free proteomics data preprocessed with MaxQuant. *Journal of proteome research* **19**: 204-211.

Sikkema L, Strobl DC, Zappia L, Madissoon E, Markov NS, Zaragosi L-E, Ansari M, Arguel M-J, Apperloo L, Becavin C. 2022. An integrated cell atlas of the human lung in health and disease. *bioRxiv*: 2022.2003. 2010.483747.

Vermeulen CJ, Xu C-J, Vonk JM, Ten Hacken NH, Timens W, Heijink IH, Nawijn MC, Boekhoudt J, van Oosterhout AJ, Affleck K. 2020. Differential DNA methylation in bronchial biopsies between persistent asthma and asthma in remission. *European Respiratory Journal* **55**.

Vieira Braga FA, Kar G, Berg M, Carpaij OA, Polanski K, Simon LM, Brouwer S, Gomes T, Hesse L, Jiang J. 2019. A cellular census of human lungs identifies novel cell states in health and in asthma.
